# Low-frequency flicker noise in a MSM device made with single Si nanowire (diameter ≈ 50 nm)

**DOI:** 10.1186/1556-276X-8-165

**Published:** 2013-04-10

**Authors:** Sudeshna Samanta, Kaustuv Das, Arup Kumar Raychaudhuri

**Affiliations:** 1 Department of Condensed Matter Physics and Material Sciences, S N Bose National Centre for Basic Sciences, Block JD, Sec 3, Salt Lake, Kolkata, 700098, India

**Keywords:** Flicker noise, MSM device, Single Si NW

## Abstract

Low-frequency flicker noise has been measured in a metal-semiconductor-metal (MSM) device made from a single strand of a single crystalline Si nanowire (diameter approximately 50 nm). Measurement was done with an alternating current (ac) excitation for the noise measurement superimposed with direct current (dc) bias that can be controlled independently. The observed noise has a spectral power density ∝1/*f*^*α*^. Application of the superimposed dc bias (retaining the ac bias unchanged) with a value more than the Schottky barrier height at the junction leads to a large suppression of the noise amplitude along with a change of *α* from 2 to ≈ 1. The dc bias-dependent part of the noise has been interpreted as arising from the interface region. The residual dc bias-independent flicker noise is suggested to arise from the single strand of Si nanowire, which has the conventional 1/*f* spectral power density.

## Background

Exploring the fundamental properties of an individual silicon nanowire (Si NW) is important as it forms the backbone of the fabrication of single-nanowire nanoelectronic devices. There are reports on the development of Si NW-based nanoscale devices such as field-effect transistors (FETs) [1,2] with wrap-around gates, surface-gated sensitive chemical and biomolecular sensors [3,4], as well as nanoscale opto-electronic devices [5]. In the context of such nanowire-based device, one important physical parameter is the low-frequency flicker noise, which has a direct impact on the device performance. In recent publications, it has been argued that flicker noise in qubits can lead to decoherence and can be the limiting factor in increasing the coherence time [6]. While flicker noise in a sub-micron metal oxide semiconductor field-effect transistor (MOSFET) with varying channel width has been investigated for some time [7], there are no reports of measurements of the low-frequency flicker noise in Si NWs and nanowire-based devices particularly with diameters much less than 100 nm.

In this paper, we report the measurement of flicker noise in a metal-semiconductor-metal (MSM) device made from a single strand of a Si NW. In such a device, the flicker noise can come from the junction region where the metals make contacts with the semiconductor (MS junction) as well as from the single Si NW. The noise arising from the junction region can be large and can even mask the noise from the Si NW by a few orders. This is because the flicker noise is likely to arise from charge carrier density fluctuations due to trapping-detrapping in the junction region. By an innovative application of direct current (dc) bias (used for biasing the device) mixed with an alternating current (ac) bias (used for the noise measurements), we could suppress the noise from the junction region and observe the noise which likely arises from the single Si NW. The enabling physics that leads to suppression of the noise in the junction region on application of the dc bias is the collapse of the depletion region at the junction region by the applied dc bias.

The low-frequency flicker noise in most materials has a power spectral density (PSD) with 1/*f* frequency dependence and can serve as a diagnostic of the presence of structural defects arising from mobility fluctuations. In semiconductors, the 1/*f* noise can also arise from recombination-generation process [8]. For the Si NW devices, proper estimation of the generic noise arising from nanowire itself is an essential device parameter for the better performance of low-noise electronics. The fluctuations in this cases arise from resistance fluctuations in a current biased system which shows up voltage fluctuations with PSD *S*_*V*_(*f*).

**Figure 1:**
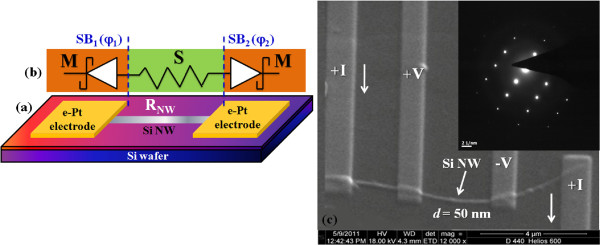
**Schematic diagram, MSM structure and SEM image.** (**a**) Schematic diagram of a single Si NW with e-beam-deposited Pt contact electrodes. (**b**) A representative MSM structure of the NW device, consisting of two Schottky diodes connected back to back with a series resistance *R*_NW_. (**c**) SEM image of the single Si NW device with four electrical leads, and the inset shows a HRTEM image of the wire itself.

Measurement of the 1/*f* noise on a single Si NW has a number of challenges that involve fabrication and manipulation of a single NW, and aligning and connecting NW with low-resistance contacts. We measured the noise in the configuration where two metal electrodes have been fabricated by nanolithography on a single Si NW. A schematic diagram of the Si NW-based device and the corresponding MSM structure are depicted in Figure 1a,b, respectively. For most of the devices, including opto-electronic devices, fabricated on a single Si NW, the basic configuration is the MSM configuration. In such cases, the contact resistance at the Schottky junction plays an important role in carrier transport through the NW. This can also lead to a substantial flicker noise at the junction regions due to the existence of traps in the depletion region. In this report, we show the noise measurement carried on with an ac excitation (*V*_ac_) with a superimposed independent dc bias ((*V*_dc_), more than the Schottky barrier height (*ϕ*) formed at the metal-semiconductor (MS) junction region) which can lead to severe suppression of the noise arising at the junction region by few orders of magnitude. This suppression of the junction noise enables us to estimate of the noise arising from the single Si NW. In the case of a single Si NW MSM device, such experiments do not exist, and the report here may provide an independent tool to reduce the junction noise by applying an external dc bias.

## Methods

### Synthesis and device fabrication

The Si NWs used in this experiment were fabricated by metal-assisted chemical etching [9] technique. The method leads to a dense array of single crystalline Si NWs with a diameter ranging from approximately 20 to 100 nm and lengths of more than 10 µm. A high-resolution transmission electron microscope (HRTEM) image shows the probable existence of an oxide layer with a thickness ≤ 2 nm at the surface. The Pt contacts (in the configuration of the MSM device) for the noise measurement were made by using e-beam-assisted local deposition of methylcyclopentadienyl platinum trimethyl precursor at a bias of 15 kV in a dual beam system FEI-HELIOS 600 (FEI Co., Hillsboro, OR, USA). The scanning electron microscopy (SEM) image of a single NW connected with four electrical contacts is shown in Figure 1c. The four electrical contacts allow us four-probe measurements of the resistance of the individual NW and hence its resistivity (*ρ*). The inner two electrodes were used for current-voltage (*I* − *V*) measurements in the MSM device configuration. Pt in a dual beam machine can also be deposited using Ga ions. However, to avoid damage as well as contamination from implanted Ga ions, we used e-beam-assisted deposition. We note that the Pt deposited from the decomposition of the high carbon-containing precursor is not pure Pt. Instead, it is a composite of carbon and Pt, which has been analysed before by our group for its physical characteristics and compositional details [10].

### Electrical measurements

The metallic contacts at the ends lead to the Schottky barrier (SB) formation in the junction region (see Figure 1b). The resulting MSM device can be modelled as two back-to-back Schottky diodes (SB_1_ and SB_2_) at the ends with a Si NW with resistance *R*_NW_ connecting them. The current passing through such a device is mainly controlled by the barrier heights *φ*_1_ and *φ*_2_ at the two contacts SB_1_ and SB_2_, respectively. This device configuration also enabled us to do two-probe as well as four-probe measurements on the same Si NW, which then allows us to find the contact resistance *R*_C_, an important device parameter. The area of contact, *A*_C_, can be obtained from the SEM image of a given device from which a reliable estimate of specific contact resistivity *ρ*_C_ = *A*_C_*R*_C_ can be obtained.

**Figure 2:**
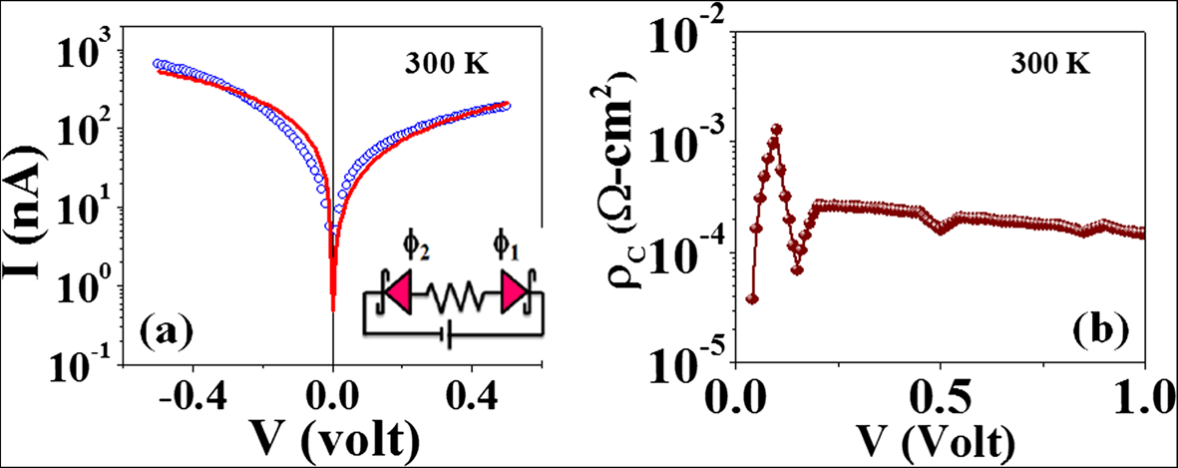
***I − V* characteristics and specific contact resistance.** (**a**) The *I* − *V* characteristics at 300 K where the solid line shows a fitted curve using Equation 1 (see text). (**b**) The variation of specific contact resistivity with bias voltage.

Figure 2a shows the non-linear and asymmetrical *I* − *V* characteristics of a typical device made from a single Si NW with diameter of approximately 50 nm. At the highest device current of 10 µA, the current density is ≈ 2.5 ×10^4^ A/cm^2^, which is much less than the electromigration damage threshold. The nanowire used has a resistivity at room temperature *ρ*_300K_ = 290 m *Ω*.cm. Comparison of the *ρ* with the resistivity of bulk Si gives us an estimate of carrier density *n* ≈ ×10^17^/cm^3^. The non-linearity at low bias is a signature of the Schottky-type contacts. The asymmetric nature of the *I* − *V* curves arises because of *φ*_1_ ≠ *φ*_2_. This inequality arises from the likely differences in the surface conditions at the two contacts (M-S) that will determine the actual value of the barriers. The bias-dependent current *I* has been fitted with the equation for back-to-back Schottky diodes connected by a resistor [11]

(1) $$\begin{array}{lll} & \;\;I(V)=I_{0}\text{exp}(\frac{qV^{\prime }}{\mathrm{\eta kT}}-1)\times \;\\ & \frac{\text{exp}(\frac{-q(\varphi_{1}+\varphi_{2})}{\text{kT}})}{\text{exp}(\frac{-q\varphi_{2}}{\text{kT}})+\text{exp}(\frac{-q\varphi_{1}}{\text{kT}})\text{exp}(\frac{qV^{\prime }}{\mathrm{\eta KT}})}\; & \; \end{array}$$

where *V*^′^ = *V* − *IR*_NW_, *R*_NW_. (In the equation above, *φ*_1_ is related to the terminal with *V*+ve.) *I*_0_ arises from thermoionic emission. The *I* − *V* data at low bias (< 0.5 V) as well as the fit to the data are shown in Figure 2a (solid line). Equation 1 fits the *I* − *V* data well, and we could obtain the barrier heights. For the data shown in Figure 2a, *φ*_1_≈ 0.1 eV and *φ*_2_≈ 0.04 eV. From the contact resistance *R*_C_ measured as a function of bias, as depicted before, we obtained the bias-dependent specific contact resistance *ρ*_C_ in Figure 2b. With increase of bias, *ρ*_C_ is substantially reduced (by nearly a factor of 2). We limited the analysis to bias up to 1 V, because the variation of *ρ*_C_ saturates for bias that is much higher than the barrier heights *φ*_1_ and *φ*_2_.

### Low-frequency noise measurements on MSM device

**Figure 3:**
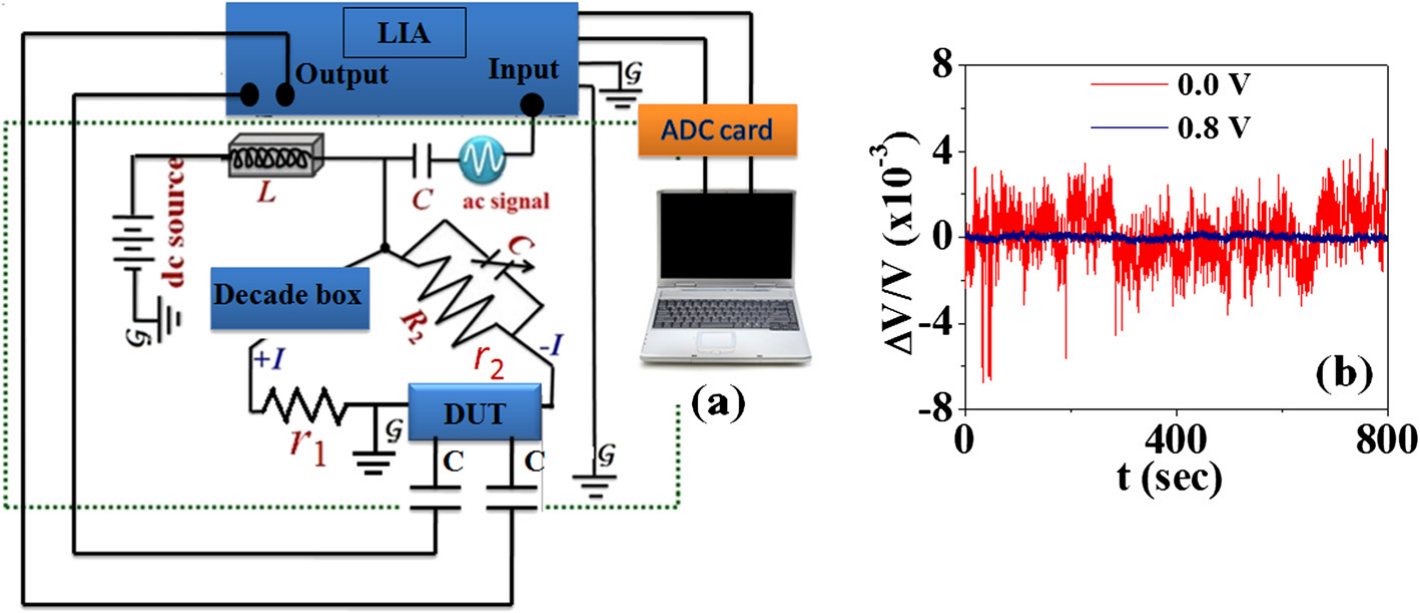
**Noise detection scheme and time series of resistance fluctuations.** (**a**) The schematic diagram of the ac noise detection scheme with the application of dc bias. (**b**) The typical time series of resistance fluctuations for two representative dc bias voltages but with the same *V*_ac_.

Measurement of low-frequency noise (resistance fluctuation) at room temperature (300 K) was done using the ac detection scheme [12] shown in Figure 3a. The ac bias *V*_ac_ is used to measure the fluctuation, while the dc bias *V*_dc_ was applied independently for tuning the device at a given point on the *I* − *V* curve [13-15]. The applied *V*_dc_ lowers the contact resistance as well as the noise from the junction region. The separate control of the *V*_ac_ and *V*_dc_ is important because it decouples the biasing needed for sending current through the MSM device from the noise measurement. Our measurement allows us, even at a relatively high level of *V*_dc_, to maintain *V*_ac_ at a low level such that $S_{V}(f)\propto V_{\text{ac}}^{2}$. This makes the noise measurement process ohmic, and one can obtain the correct value of the relative fluctuations. The noise spectra were taken in the window *f*_min_ = 0.01 Hz to *f*_max_ = 10 Hz. The normalized variance of resistance noise (mean square fluctuation) can be obtained as $\langle {\left(\mathrm{\Delta R}\right)}^{2}\rangle /R^{2}\equiv (1/V_{\text{ac}}^{2})\int_{f_{\min}}^{f_{\max}}S_{V}(f)\text{df}$, where *f*_min_ → *f*_max_ is the bandwidth of measurements. For *f* > *f*_max_, background noise (mostly Nyquist noise) dominates, and for *f* < *f*_min_, long-term drifts interfere with the measurement because of long data acquisition time [15]. The magnitude as well as the PSD shows a large dependence on the dc bias. Figure 3b shows the typical time series of resistance fluctuations for two representative dc bias voltages but with the same *V*_ac_.

The noise data reported here were taken with the contact with larger barrier height (*φ*_1_) forward biased. The dominant contribution to the contact noise as well as the contact resistance arises from this contact. On applying forward bias to this junction, the noise (as well as the contact resistance) is severely reduced. The other contact with much smaller barrier (*φ*_2_) has much less contribution to the contact noise. Thus, even if it is reversed biased (and the depletion width increases due to the reverse bias), its contribution still remains low.

## Results and discussion

**Figure 4:**
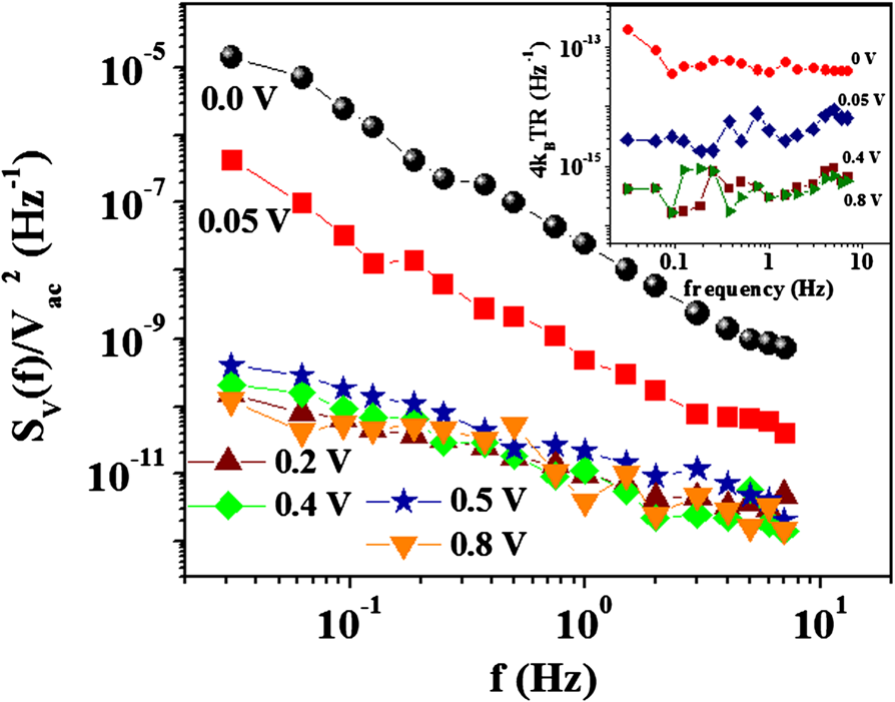
**The power spectral density**$S_{V}(f){/V}_{\mathrm{ac}}^{2}$**as a function of frequency*f* at few representative superimposed*V***_**dc**_**.** The inset shows the Nyquist noise for different *V*_dc_.

The normalised PSD $S_{V}(f)/V_{\text{ac}}^{2}$ is shown in Figure 4 which is ∝ 1/*f*^*α*^. The data has been taken with varying dc bias. The superimposed dc bias reduces the magnitude of $S_{V}(f)/V_{\text{ac}}^{2}$, and the change is approximately five orders of magnitude. The dc bias also changes the nature of frequency dependence. For *V*_dc_ = 0, *α*≈2. However, *α* becomes approximately 1 for *V*_dc_ ≥ 0.2 V, which is larger than the barrier heights. Thus, the applied *V*_dc_ reduces not only the specific contact resistance *ρ*_C_, but also the magnitude of PSD significantly along with a change in the frequency dependence. Our measurement also allows independent measurement of the frequency-independent background noise *S*_bg_. The inset of Figure 4 shows the *S*_bg_ with different applied *V*_dc_. We find that *S*_bg_ is also reduced with increased *V*_dc_, although it is much less than the suppression of the flicker noise. The *S*_bg_ was found to be the same as the Nyquist noise *S*_nyq_ = 4*k*_*B*_*TR*, where *R* is the total resistance = *R*_C_ + *R*_NW_. The reduction of the Nyquist noise occurs mainly due to reduction of *R*_C_ by the dc bias. This analysis separates out the noise due to the contact resistance which appears in the frequency-independent Nyquist noise. The observed flicker noise (*S*_*V*_(*f*)) occurring on top of the Nyquist noise has two components: one arising from the junction region at the M-S interface and the other likely from the bulk of the Si NW. This can be intrinsic for the NW and can arise either from the defect-mediated mobility fluctuation or the carrier density fluctuation which arises from recombination-generation process [16]. The superimposed bias *V*_dc_ dependence of the flicker noise cleanly separates out the above two contributions.

**Figure 5:**
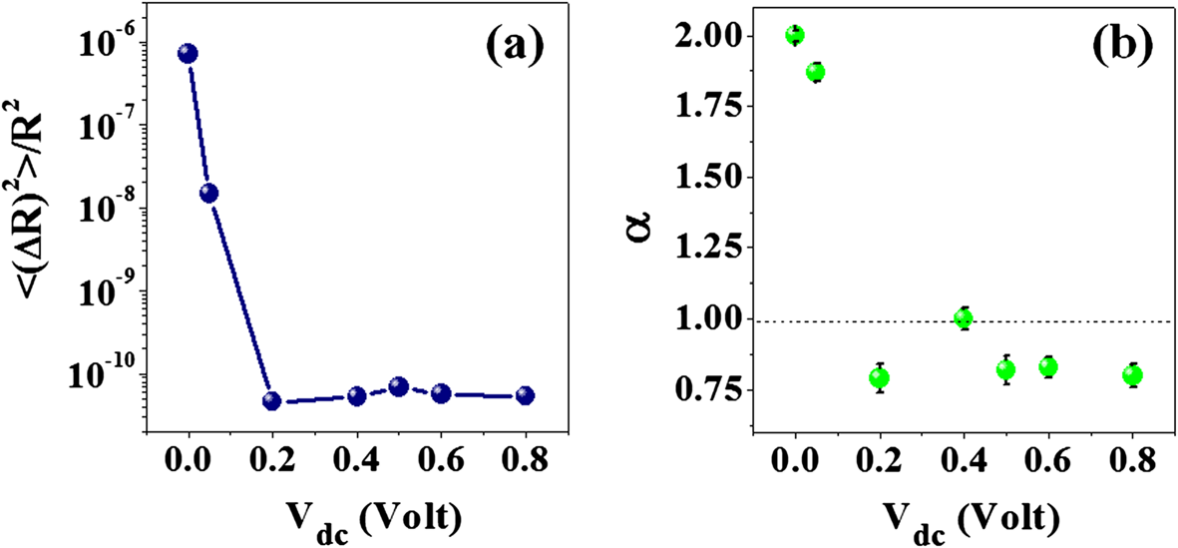
**The variation of (a)* 〈(ΔR)***^***2***^***〉 / R***^***2***^** and (b)* α* as a function of*V***_**dc**_**at 300 K.**

To elucidate further, we have plotted the normalized mean square fluctuation 〈(*ΔR*)^2^ 〉/*R*^2^ as a function of *V*_dc_ in Figure 5a. There is a steep decrease of 〈 (*ΔR*)^2^ 〉/*R*^2^ by more than four orders, when *V*_dc_ > 0.2 V. At low *V*_dc_ (< barrier height), the noise is predominantly dominated by the junction noise. For higher *V*_dc_, the junction noise is suppressed substantially, and residual observed noise gets dominant contribution likely from the intrinsic noise due to the Si NW. The changing spectral character of PSD is quantified by *α* plotted against *V*_dc_ in Figure 5b. We found that *α* is nearly 2 for low *V*_dc_ and can arise from the depletion region at the M-S contact. For *V*_dc_ > 0.2 V, *α* decreases and reaches a bias-independent value of 0.8 ± 0.1. *α* ≈ 1 is an indication of conventional 1/*f* noise spectrum which arises from the Si NW.

Evaluation of the noise in a single Si NW needs to be put in perspective and compared with bulk systems. In noise spectroscopy, one often uses a quantitative parameter for noise comparison is the Hooge parameter [17]. The spectral power of 1/*f* noise in many conductors often follows an empirical formula [17]$S_{V}(f)=\frac{\gamma_{H}V_{\text{ac}}^{2}}{\mathrm{N.}f^{\alpha }}$ where *γ*_H_ is the Hooge’s parameter, and *N* is the number of carriers in the sample volume (between voltage probe leads). *γ*_H_ is a useful guide when one compares different materials. Usually, a low *γ*_H_ is associated with a sample with less defect density that contributes to the 1/*f* noise arising from the defect-mediated mobility fluctuation [18]. *N* can be calculated from carrier density *n* = 2×10^17^/cm^3^ and volume of the sample between two voltage leads (30×10^−16^ cm^3^). Our Si NW under test has *N* ≈ 600. We obtained *γ*_H_ ≃ 10^ − 8^ when we use the limiting value of the PSD for *V*_dc_ ≥ 0.2 V.

For bulk crystalline Si, the noise has been studied extensively both in low-doped and degenerately doped crystals [15] as well as in films [19,20]. In bulk Si wafers with low doping concentration, the value of *γ*_H_ lies in the range of 10^ − 7^ to 10^ − 2^ with the exact value being a sensitive function of impurity and defect process conditions [15,17]. For the Si NW, we observed that the value can even be lower. We note, however, that in this size range, it has not been established that such a scaling of spectral power with 1/*N* truly holds as there can also be significant surface contributions. Thus, the use of *γ*_H_, as a parameter for comparison is done with caution. The intrinsic contribution in a NW can be large because *N* is small. In a NW, if the *γ*_H_ is indeed low as observed, this will mitigate the increase in the intrinsic noise on size reduction. For even smaller devices with smaller diameter, less dopant and closer contacts, *N* can even be below 10.

In this report, we propose a likely scenario of suppression of the junction noise by *V*_dc_. The noise at the M-S contact can arise in the depletion region where the SB forms. The traps in the depletion region can lead to substantial noise due to trapping-detrapping of carriers. Such a noise has been observed also in the depletion region of MOSFETs [7]. Flicker noise in sub-micron MOSFETs [7] have been investigated experimentally as well as theoretically, and it shows the existence of both 1/*f*^2^ and 1/*f* frequency components, where the 1/*f*^2^ component arises from charge exchange with traps in the oxide region. Application of the dc bias reduces the depletion width (*d*_dw_). In an ideal SB, *d*_dw_ ∝ (*ϕ* − *V*_dc_)^1/2^; for *V*_dc_ ≥ *ϕ*, *d*_dw_→0. In such case, the trapping centres are occupied and cannot contribute to the trapping-detrapping process generated noise. This leads to severe suppression of the noise in the junction region. Another strong evidence that the noise at the junction arises from the trap states in the depletion region is the value of the exponent *α*. It has been shown that existence of trap states in the depletion region can lead to a power spectrum of the type *S*_*v*_(*f*) ∝ 1/*f*^*α*^ where *α* = 2 [21]. We also found *α* ≈ 2 for a very low dc bias, when the observed noise is mainly due to the junction noise. *α* rapidly reduces to ≈ 1 for high *V*_dc_. The suggested mechanism for noise reduction with applied *V*_dc_ is the controlling of *d*_dw_ which can be a generic mechanism for an MSM device and thus has a general applicability for such junctions.

## Conclusion

To summarize, we have measured the electrical noise in an MSM device consisting of a single stand of Si NW with a diameter of approximately 50 nm. The flicker noise as well as Nyquist noise was measured with ac excitation with a superimposed dc bias. On application of a dc bias, more than the Schottky barrier height reduces the contact resistance which leads to reduction of the Nyquist noise as well as the flicker noise (∝ 1/*f*^*α*^) in the MSM device along with a change of *α* from 2 to ≃1. The part of the noise suppressed by dc bias has been interpreted as arising due to trapping-detrapping noise in the depletion region at the interface. The residual noise has been has been linked to the noise in the single Si NW, which has the conventional 1/*f* spectral power density with an estimated Hooge parameter *γ*_H_ ≃ 10^ − 8^.

## Abbreviations

HRTEM: High-resolution transmission electron microscopy.

## Competing interests

The authors declare that they have no competing interests.

## Authors’ contributions

KD synthesized the Si NWs and fabricated the single NW device by nanolithography. SS did all the electrical measurements and the low-frequency noise measurements. SS performed the treatment and calculations on the experimental data and prepared the manuscript initially. AKR gave sufficient ideas and concepts to the whole work. All authors have read and approved the manuscript.
